# Can mass drug administration alone eliminate lymphatic filariasis in areas of Indonesia with zoophilic *Brugia malayi*?

**DOI:** 10.1371/journal.pntd.0014501

**Published:** 2026-07-14

**Authors:** Taniawati Supali, Yenny Djuardi, Elisa Iskandar, Noviani Sugianto, Rahmat Alfian, Yossi Destani, Emanuele Giorgi, Gary J. Weil, Peter U. Fischer

**Affiliations:** 1 Department of Parasitology, Faculty of Medicine, Universitas Indonesia, Jakarta, Indonesia; 2 Centre of Health Informatics, Computing, and Statistics, Lancaster University Medical School, Lancaster, United Kingdom; 3 Infectious Diseases Division, Department of Medicine, Washington University School of Medicine, St. Louis, Missouri, United States of America; NEHU: North Eastern Hill University, INDIA

## Abstract

Belitung district, Indonesia, is endemic for lymphatic filariasis (LF) caused by zoophilic *Brugia malayi.* The district received 5 rounds of mass drug administration (MDA) with diethycarbamazine and albendazole (DA) between 2006 and 2010 and passed three transmission assessment surveys (TAS). Post-TAS-3 surveillance in 2021 revealed a prevalence of microfilariae (Mf) of 2.1%. This led to district-wide resumption of MDA with co-administered ivermectin, diethylcarbamazine, and albendazole (IDA). The impact of two rounds of IDA MDA was assessed using a modified IDA Impact Survey design. At baseline survey in 2022, we tested 5,161 adults in 30 clusters drawn from 26 villages. The overall Mf prevalence was 1.57% (95% CI 1.25 – 1.95), although Mf-positive individuals were only detected in 16 villages. Based on geospatial predictions, follow-up surveys were conducted in 12 villages including 1 village that was previously not chosen in the baseline randomization. Further analysis focused on the 11 villages that were surveyed three times. The overall Mf prevalence in adults at the baseline was 2.51% (95% CI 1.99 – 3.14). Prevalence of Mf carriers after one and two rounds of MDA was significantly decreased compared to the baseline (0.86% (95% CI 0.59 – 1.21) and 1.10% (95% CI 0.78 – 1.49)) respectively. Follow-up surveys showed that IDA was effective for clearing Mf, and more than 90% of the individuals in the follow-up surveys reported consumption of at least one IDA treatment. However, we detected new Mf-positive individuals during each of the follow-up surveys. In this setting, two rounds of IDA were insufficient to eliminate *B. malayi*. Areas with zoophilic *B. malayi* may need to be designated by WHO as special intervention zones for LF elimination that require additional MDA and more intense post MDA surveillances.

## Author summary

Belitung district in Indonesia, known as an area endemic for zoophilic *Brugia malayi*, received mass drug administration (MDA) with diethylcarbamazine and albendazole from 2006 to 2010. A surveillance in 2021 revealed a microfilaria (Mf) prevalence of 2.1% in adults, prompting the reintroduction of MDA with co-administered ivermectin, diethylcarbamazine, and albendazole (IDA). The Mf prevalence in school-aged children was low and the impact of two rounds of IDA MDA was assessed using modified IDA Impact Survey of adults. At baseline we surveyed 26 villages, and 81 (1.57%) Mf-positive individuals were detected. The Mf prevalence in the same 11 villages that were examined at baseline, first and second follow-ups was 2.51%, 0.86%, and 1.1%, respectively. Although IDA treatment was still effective in clearing Mf, we continued detecting new Mf-positive individuals in the follow-up surveys. In this setting, two rounds of IDA were insufficient to eliminate *B. malayi*, therefore this area with potential zoonotic *B. malayi* transmission may require additional strategies to WHO recommendation, such as intensified surveillance and MDA.

## Introduction

*Brugia malayi* is the predominant filarial species that causes lymphatic filariasis (LF) in Indonesia, and it is present in 159 of 236 (67%) endemic districts. *B. malayi* occurs in zoophilic and anthropophilic strains. The two types are differentiated by sheath shedding behavior, periodicity patterns, mosquito vector species and by their presence in animals [1–3]. It has been reported decades ago that domestic and wild animals such as cats, dogs, and monkeys (*Macaca fascicularis, Presbytis spp.*) were naturally infected with zoophilic *B. malayi* and may serve as animal reservoirs. For example, in a village in Sumatra 7% of the cats and 20% of the macaques were found to be infected with *B. malayi* and another study showed that leaf monkeys were more susceptible to infection than macaques [4,5]. However, most studies on the animal reservoir of *B. malayi* were conducted in the last century when monkeys were more abundant and many studies did not differentiate between *B. malayi* and the animal parasite *Brugia pahangi* [6].

Belitung district in western Indonesia is known to be endemic for zoophilic *B. malayi* [7,8]. The district received five rounds of mass drug administration (MDA) with diethylcarbamazine (DEC) and albendazole (DA) between 2006 and 2010 and passed three transmission assessment surveys (TAS) between 2011 and 2016. The TAS surveys assessed the prevalence of anti-filarial antibodies in young children without testing adults. The TAS survey results led Indonesia’s national LF elimination program to classify Belitung as an area with interrupted transmission of LF (S1 Fig). However, post-TAS-3 surveillance surveys eleven years after the last round of MDA documented Mf prevalences in adults between 1% and 5.9% in several villages [9,10]. These survey results were alarming for district health authorities and for Indonesia’s national LF elimination program. This led to a plan to resume MDA according to WHO guidelines with the triple drug regimen IDA (ivermectin plus DEC and albendazole [11,12].

Several randomized clinical trials showed that IDA was superior to DA for clearing *Wuchereria bancrofti* and *Brugia timori* Mf [13,14]. In addition, a large cohort event monitoring study on over 26,000 people in five countries including Indonesia showed that IDA is as safe as DA for MDA [15]. Furthermore, in our previous study in Belitung, we administered IDA to 35 people infected with *B. malayi*, and 94% were free of Mf one year after treatment [10]. While the efficacy and safety of IDA for MDA have been established and many millions of people have received MDA in areas endemic for *W. bancrofti* [16], experience with IDA MDA in areas with zoophilic *B. malayi* is limited.

For areas that failed TAS or show resurgence of infection during surveillance, WHO recommends at least two rounds of effective MDA. Therefore, the aim of the present study was to assess the impact of two rounds of IDA MDA on Mf prevalence of zoophilic *B. malayi* in the human population. The study covered a large implementation unit which had persistent or resurgent LF following MDA with DA. We used the WHO recommended IDA Impact Survey study design to plan our study and used an updated sampling method for cluster selection based on Mf prevalence and geospatial data.

## Methods

### Ethics statement

This study received ethical approval from the ethical committee of Universitas Indonesia no. 515/UN2.F1/ETIK/PPM.00.02/2022. Oral informed consent was obtained from each participant over the age of 17. For participants under or equal to the age of 17, the oral consent was provided by their parents or guardians. Children and teenagers were registered at school and sampled at night in the community after oral consent was obtained. In rural Indonesia oral consent for finger prick blood collection is well accepted and residents know the procedure well from the testing for malaria. Written consent was only obtained for the 12 adults who provided repeated capillary blood samples for Mf periodicity testing.

### Study area

Belitung Island (part of the Sumatra region) was identified as an endemic area for LF in 2004 (S1 Fig). The island is divided into two districts, Belitung and Belitung Timur. The study was conducted in Belitung district, which included 49 administrative villages and approximately 190,000 inhabitants in 2021 [17]. The majority of the population lives in the 7 urban villages (kelurahan) of the district capital, Tanjung Pandan, which forms the biggest city on the island and harbors the main airport. There were nine primary health centers in the district. The people living inland usually work in plantations, cultivating pepper or palm and mining tin, while those along the coast make their living as fishermen. The extensive cultivation of palm trees has transformed the environment, including the natural habitat of monkeys (*Macaca fascicularis*) that are potential reservoirs for *B. malayi*. As a result, the monkeys have started entering residential areas. In addition, mining activities in the rural areas have degraded the environment, because unused mining pits accumulate water and can serve as mosquito breeding sites.

### Sampling design

WHO has recommended a special approach for sampling adult populations to monitor the impact of IDA MDA. This approach, known as the IDA Impact Survey (IIS)–Cluster Sampling Design, has two versions depending on whether the LF vector is *Aedes* or non-*Aedes*. In this study, the IIS for non-*Aedes* was used to collect baseline data before implementing MDA with IDA [18,19]. The IIS program assists researchers in randomly selecting 30 clusters, corresponding to 30 villages, using probability proportional to estimated size (PPES). For the baseline survey, we used a list of 49 administrative villages along with their total population. The program also determined the number of samples required for each cluster (S1 Table).

Four urban villages, each with one cluster, were initially selected but had to be excluded from the selection because they were located in the district capital, Tanjung Pandan, such as in the airport, harbour or government offices and residences. These areas are not known to be endemic for LF [20]. To replace these excluded villages, an additional cluster from each of four villages—Air Seruk, Badau, Lassar, and Membalong—was added. While most of the selected villages had a total population of less than 3,000 residents, these four villages had more than 3,000 residents; therefore they have larger sample size (regarded as 2 clusters).

For exploratory reasons, an additional survey on workers of a palm oil plantation was also done at baseline, and another cluster in a plantation was surveyed in the first follow-up. The majority of the population in the plantations were migrant workers from other Indonesian islands who stayed for only 2–3 years on the plantation.

To monitor the efficacy of IDA after the first and second rounds of MDA, we used geostatistical analysis and cluster selection methods developed by a co-author (EG). A binomial geostatistical model [21], with an intercept-only specification, was used to analyse the prevalence of Mf. This approach accounts for spatial correlation in reported cases through a stationary and isotropic Gaussian process with an exponential correlation function (S1 Text). The use of geostatistical methods allows us to estimate LF prevalence at any location and assess the probability of exceeding a 1% Mf prevalence threshold. The model was implemented using the PrevMap package in the R software environment [22].

Based on the fitted binomial geostatistical model, we computed the probability that Mf prevalence exceeds 1% in unsampled villages. This exceedance probability metric serves as an indicator for identifying villages with higher levels of LF transmission. Subsequently, we ranked the unsampled villages based on their exceedance probability and selected the top 30 clusters with the highest likelihood of surpassing the 1% prevalence threshold.

### Ivermectin, DEC, and Albendazole (IDA) treatment

Although Belitung district had already passed three consecutive Transmission Assessment Surveys (TAS) in schoolchildren, WHO recommended that additional MDA with IDA for two consecutive years should be used in areas with persistence or recrudescence [14,18]. The Indonesian Ministry of Health recommended directly observed, height-based dosing of IDA MDA. MDA was performed in Belitung by village health workers (cadres) in October–December 2022 and October–December 2023. In both years, the majority of people were treated in October with a mop-up in November and early December.

### Sample collection

At baseline, finger prick night blood samples were collected in June 2022. Following the first and second MDA IDA treatment, samples were collected in June 2023 and June 2024. In Indonesia, several households make up a household group (called Rukun Tetangga/RT). Each village consists of several household groups depending on the total population. The sample collection was randomly based on households in the household groups. For instance, in a village with 25 household groups, the team collected 1 cluster (110 samples) from all 25 RTs. Therefore, a collection of 4–5 samples per RT was targeted. For the 4 villages with 2 clusters, 2 times 110 samples were collected.

After explaining the purpose of the study, name, age, gender, and address of the participants were recorded. During the follow-up surveys, compliance with MDA was asked of each adult participant using a questionnaire. The assessed compliance was compared to treatment coverage reported by the local health center for a plausible cross-check.

### Periodicity of *B. malayi* Mf

**Fig 1:**
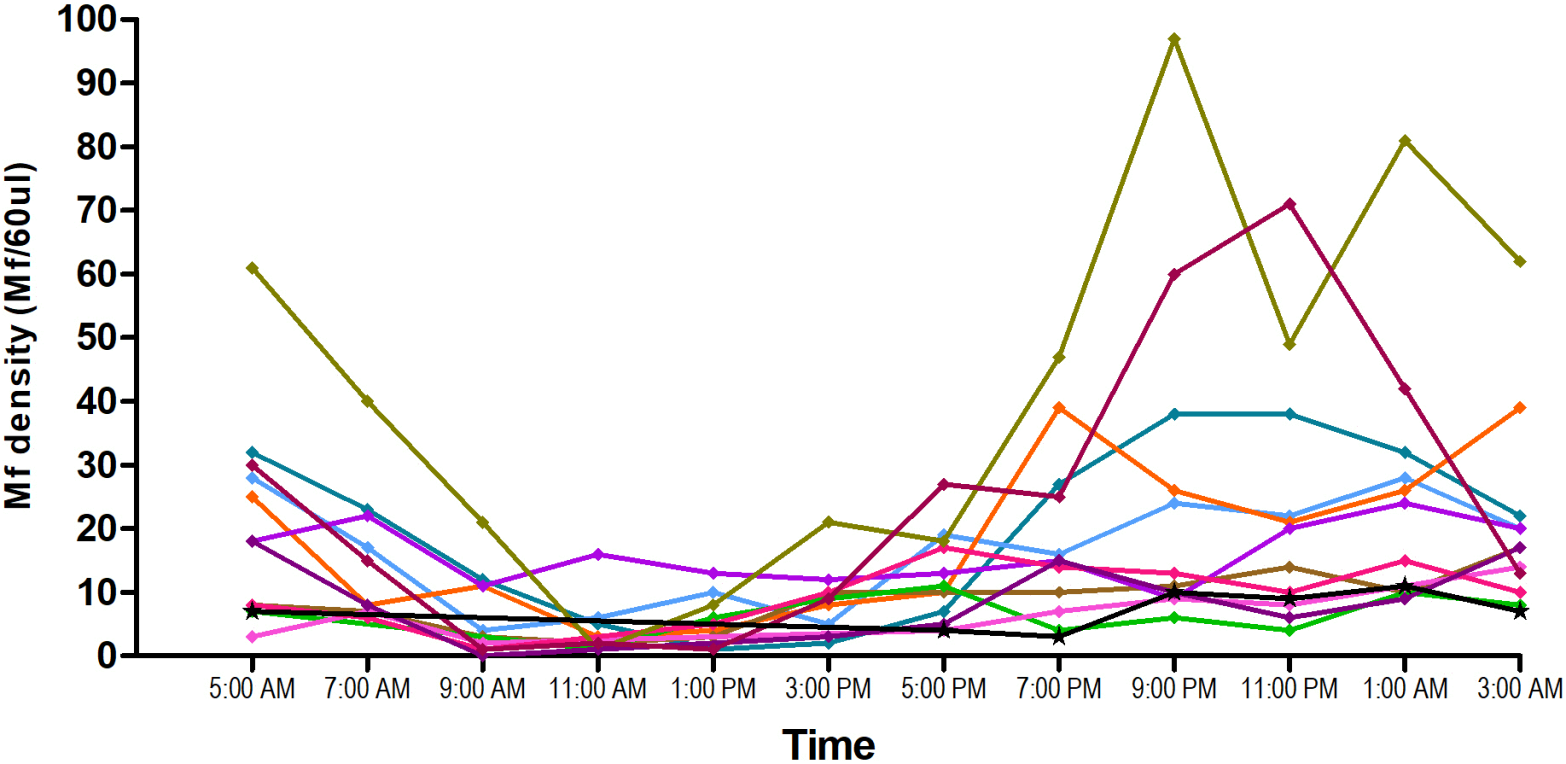
Periodicity of *B. malayi* microfilariae (Mf) in humans. Capillary blood was collected from 12 microfilaremic individuals (each depicted with different colour of line) every 2 hours. Mf were counted by microscopy of Giemsa stained three-line blood smears (60 µL).

Considering the environmental factors and the detection of *B. malayi* infected monkeys in Belitung district, the area is believed to be endemic for the zoophilic type of *B. malayi* [7]. Therefore, we decided to perform 24-hour blood draws from 12 Mf-positive individuals who were identified as Mf-positive during the preliminary survey and resided in three villages (Kembiri, Lassar, and Selat Nasik) [9]. The purpose of taking blood every two hours for a total of twenty-four hours was to determine the *B. malayi* periodicity and to plan the collection time for capillary blood accordingly. The result showed Mf were detected constantly between 5:00 PM and 5:00 AM with the highest between 07:00 PM and 11:00 PM. The periodicity pattern of Mf was classified as nocturnally sub-periodic (Fig 1). These data did not only characterize the local strain of *B. malayi* but also determined the optimal time for blood collection for this study, which started at 07:00 PM.

### Night blood collection and thick blood smear

Night blood samples were collected from 07:00 PM–11:00 PM in each selected village. Finger prick blood samples were collected from each participant. A total of 250 µL of blood was stored in an EDTA microtainer labelled with a unique barcode. Thick blood smears were made in the field laboratory in Tanjung Pandan.

A total of 60 µL blood was taken from an EDTA microtainer to prepare a three-line smear onto the slide. The slides were air-dried for two days, blood was hemolyzed using water, fixed with methanol, and stained with Giemsa. The slide was examined under the microscope to detect and count Mf [18].

### Statistical analysis

Collected demographic data such as name, gender, age, address (RT number and village name), and Mf count were entered into an Excel file. GraphPad Prism 5 was used to make graphs. Statistical analysis was performed using SPSS version 20. The Mf density of Mf-positive individuals who were followed up was presented as a median with interquartile range (IQR), and geometric mean with range. A chi-square test was used to assess the significance of gender differences in infection prevalence and the significance of changes in Mf prevalence following MDA. A statistical result was considered significant if the P-value is less than 0.05.

## Results

### Mf prevalence at baseline before IDA MDA

#### A. Mf prevalence in children.

To test whether children would be a suitable sentinel group, we examined 1,021 children (498 boys and 523 girls) from elementary and junior high schools in villages with Mf-positive adults known from the previous study in 2021 [10]. These children (aged between 6 and 14 years) were invited at school for capillary night blood collection. Occasionally younger or older siblings joined them with their parents for blood collection; therefore, the mean age was 11.20 years with a range between 3 and 17 years. Microscopic examination of night blood smears revealed that 6 children (aged 6, 7, 8, 9, 12 and 15 years) were positive for *B. malayi* Mf, resulting in a prevalence of 0.59% (95% CI 0.22 – 1.27) (Table 1). This Mf prevalence in children was significantly lower than the Mf prevalence in adults from the same villages (2.89%, 95% CI 2.25 – 3.64, p = 0.009).

**Table 1:** Microfilarial prevalence in children by gender at baseline in villages with adult Mf carriers.

Village	Male		Female		Total		95% CI (%)
	N	Mf prevalence (n, %)	N	Mf prevalence (n, %)	N	Mf prevalence (n, %)	
Selat Nasik	101	0 (0)	128	0 (0)	229	0 (0)	0.00–1.60
Suak Gual	37	0 (0)	46	0 (0)	83	0 (0)	0.00–4.35
Petaling	25	1 (4.00)	23	0 (0)	48	1 (2.08)	0.05–11.07
Bantan	35	1 (2.86)	35	0 (0)	70	1 (1.43)	0.04–7.70
Simpang Rusa	53	0 (0)	52	0 (0)	105	0 (0)	0.00–3.45
Lassar	183	2 (1.09)	172	2 (1.16)	355	4 (1.13)	0.31–2.86
Kembiri	64	0 (0)	67	0 (0)	131	0 (0)	0.00–2.78
All villages	498	4 (0.80)	523	2 (0.38)	1,021	6 (0.59)	0.22–1.27

### B. Mf Prevalence in adults

To determine the Mf prevalence before MDA, night blood collection was done in 30 clusters drawn from 26 villages. A total of 5,161 adults, 2,859 (55%) females and 2,302 males, participated in this survey. Their mean age was 43.5 years with a range between 18 and 95 years. The microscopic examination revealed that 81 individuals (59 men and 22 women) were Mf-positive, resulting in the Mf prevalence of 1.57% (95% CI 1.25 –1.95) (Table 2). The prevalence in males (2.56%) was significantly higher than in females (0.77%) (p < 0.0001), with a majority of Mf-positives were over 50 years of age (S2 Table).

**Table 2:** Overall Mf prevalence in adult participants by gender at baseline.

Village	Cluster	Male		Female		Total		95% CI (%)
		N	Mf prevalence (n, %)	N	Mf prevalence (n, %)	N	Mf prevalence (n, %)	
Selat Nasik	1	224	17 (7.59)	375	7 (1.87)	599	24 (4.01)	2.58–5.90
Suak Gual	1	42	3 (7.14)	71	1 (1.41)	113	4 (3.54)	0.97–8.82
Petaling	1	63	2 (3.17)	96	1 (1.04)	159	3 (1.89)	0.39–5.41
Sungai Padang	1	41	1 (2.44)	53	1 (1.89)	94	2 (2.13)	0.26–7.48
Air Batu Buding	1	49	1 (2.04)	61	0 (0)	110	1 (0.91)	0.02–4.96
Badau	2	138	2 (1.45)	172	0 (0)	310	2 (0.65)	0.08–2.31
Bantan	1	104	3 (2.88)	118	1 (0.85)	222	4 (1.80)	0.49–4.55
Simpang Rusa	1	114	1 (0.88)	185	4 (2.16)	299	5 (1.67)	0.55–3.86
Lassar	2	220	13 (5.91)	256	1 (0.39)	476	14 (2.94)	1.62–4.89
Kembiri	1	232	11 (4.74)	291	4 (1.37)	523	15 (2.87)	1.61–4.69
Tanjung Rusa	1	53	2 (3.77)	66	0 (0)	119	2 (1.68)	0.20–5.94
Padang Kandis	1	75	0 (0)	90	0 (0)	165	0 (0)	0.00–2.21
Gunung Riting	1	86	1 (1.16)	100	0 (0)	186	1 (0.54)	0.01–2.96
Membalong	2	185	0 (0)	165	0 (0)	350	0 (0)	0.00–1.05
Perpat	1	62	1 (1.61)	54	0 (0)	116	1 (0.86)	0.02–4.71
Sungai Samak	1	62	0 (0)	52	0 (0)	114	0 (0)	0.00–3.18
Cerucuk	1	49	0 (0)	65	0 (0)	114	0 (0)	0.00–3.18
Kacang Botor	1	50	0 (0)	61	0 (0)	111	0 (0)	0.00–3.27
Air Seruk	2	77	0 (0)	114	0 (0)	191	0 (0)	0.00–1.91
Batu Itam	1	59	0 (0)	54	0 (0)	113	0 (0)	0.00–3.21
Terong	1	45	0 (0)	50	1 (2.00)	95	1 (1.05)	0.03–5.73
Air Selumar	1	63	0 (0)	66	0 (0)	129	0 (0)	0.00–2.82
Pelepak Pute	1	45	0 (0)	61	0 (0)	106	0 (0)	0.00–3.42
Sijuk	1	43	0 (0)	58	0 (0)	101	0 (0)	0.00–3.59
Keciput	1	50	1 (2.00)	50	1 (2.00)	100	1 (1.00)	0.03–5.45
Mentigi	1	71	1 (1.41)	75	0 (0)	146	1 (0.68)	0.02–3.76
Total 26 villages	30	2,302	59 (2.56)	2,859	22 (0.77)	5,161	81 (1.57)	1.25–1.95

**Fig 2:**
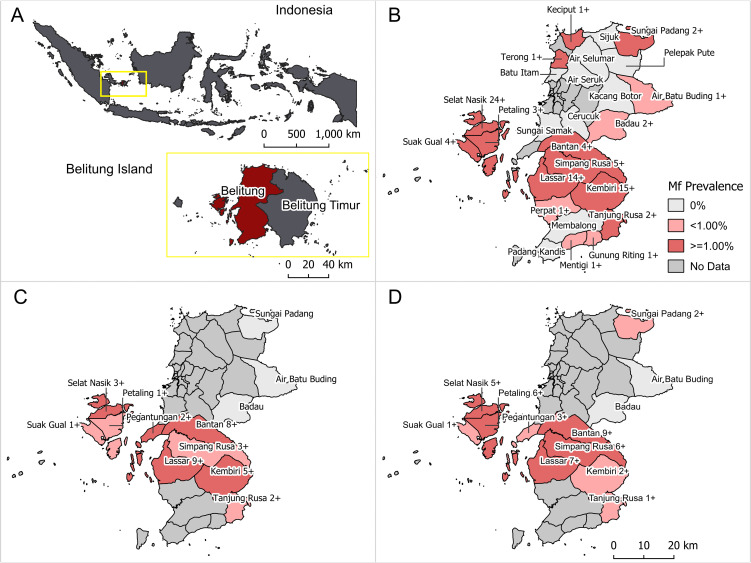
Map of selected villages with microfilaria prevalence before and after MDA. (A) Map of Indonesia showing the location of Belitung island which is divided into Belitung District and Belitung Timur District. (B) 2022: Mf-positive individuals were detected in 16 out of 26 surveyed villages. (C) 2023: Mf-positive individuals were detected in 9 out of 12 villages. (D) 2024: Mf-positive individuals were detected in 10 of 12 surveyed villages. The base map for Indonesia was sourced from GADM (https://gadm.org/download_country.html, select ‘Indonesia’) under the GADM license (https://gadm.org/license.html) and visualized using QGIS.

Microfilaremic individuals were detected in 16 villages, with Mf prevalences that ranged from 0.54% to 4.01% (Fig 2). Of those endemic villages, 11 had Mf prevalences ranging from 1.00% to 4.01% and 5 had Mf prevalences below 1% (0.54%–0.91%).

A total of 85 employees in a palm oil plantation participated in the night blood sample collection, but no Mf-positives were found. The majority of employees originated from non-endemic areas such as Lombok and Java.

### Mf prevalence after the first round of IDA MDA

Geostatistical analysis and Mf prevalence informed cluster selection for follow-up surveys. A one-year follow-up survey was conducted in 12 high-risk villages (11 villages from baseline and 1 additional village, Pegantungan, obtained during randomization by geostatistical analysis) (Fig 2 and S1 Table).

Mf-positives were detected in 9 of 12 villages and the Mf prevalence ranged from 0.47% to 2.77%, with overall prevalence 0.88% (95% CI 0.61 – 1.23) (Table 3). Five villages had a Mf prevalence > 1%. A total of 3,869 individuals, including 2,126 women and 1,743 men participated in the follow-up night blood survey. Among these, 34 participants, 26 men and 8 women, were identified as Mf-positive, and most of them were over 50 years old (S2 Table). The Mf prevalence was higher in men than women (1.49% vs 0.38%, p < 0.0001). Among Mf-positive individuals, there were two positives out of 154 samples from Pegantungan village (1.30%). When the Mf prevalence after the first round of MDA was compared to the baseline, there was a significant decrease (p = 0.004).

**Table 3:** Mf prevalence and density (median, geometric mean) before and after IDA MDA.

MDA	No. of Villages	No. of Mf +		No. examined	Prevalence (%)	Median (IQR, Mf/ml)	Geomean (range, Mf/ml)
		Old	New				
Baseline	26	81		5,161	1.57 (1.25 – 1.95)	176 (80 – 440)	197 (151 – 257)
Post-IDA Round 1	12	12	22	3,869	0.88 (0.61 – 1.23)	168 (48 – 388)	153 (96 – 242)
Post-IDA Round 2	12	15	27	3,923	1.07 (0.77 – 1.44)	144 (80 – 400)	183 (128 – 261)

Microscopic examination of blood smears from 129 palm oil plantation employees, all males, showed no Mf-positives. In total, all surveyed employees (214) in the first and second plantations were negative.

### Mf prevalence after the second round of IDA MDA

During the second follow-up, Mf-positive individuals were detected in 10 of 12 villages. The Mf prevalence ranged between 0.25% and 5%, 5 villages had a prevalence above 1% (Fig 2). A total of 3,923 individuals were screened in the second follow-up and 42 persons (32 men and 10 women) were Mf-positive. Again the Mf prevalence was higher in men (1.80%) than in women (0.47%, p < 0.0001). The total Mf prevalence was 1.07%, and significantly lower compared to baseline data (p = 0.042), but not different from the first follow-up (p = 0.389) (Table 3). The mean Mf density of Mf-positive individuals was also similar at baseline, follow-up 1 and follow-up 2 (Table 3). Meanwhile, three Mf-positive individuals were found among 364 adults in Pegantungan (0.82%).

A total of 11 villages were examined at baseline and at both follow-ups (Table 4). A significant decrease of Mf prevalence was observed between the baseline and the first round of Post-MDA follow-up (p < 0.001). In the second follow-up, total Mf prevalence was 1.10%, and significantly lower compared to baseline data (p < 0.001), but not different from the first follow-up (p = 0.309) (Table 4).

**Table 4:** Mf prevalence in 11 villages that were annually followed up after IDA MDA.

Village	Baseline		Post-IDA Round 1		Post-IDA Round 2	
	N	Mf prevalence (n, %)	N	Mf prevalence (n, %)	N	Mf prevalence (n, %)
Selat Nasik	599	24 (4.01)	270	3 (1.11)	285	5 (1.75)
Suak Gual	113	4 (3.54)	177	1 (0.56)	136	1 (0.74)
Petaling	159	3 (1.89)	106	1 (0.94)	120	6 (5.00)
Sungai Padang	94	2 (2.13)	330	0 (0)	254	2 (0.79)
Air Batu Buding	110	1 (0.91)	201	0 (0)	275	0 (0)
Badau	310	2 (0.65)	475	0 (0)	397	0 (0)
Bantan	222	4 (1.80)	289	8 (2.77)	385	9 (2.34)
Simpang Rusa	299	5 (1.67)	322	3 (0.93)	433	6 (1.39)
Lassar	476	14 (2.94)	619	9 (1.45)	582	7 (1.20)
Kembiri	523	15 (2.87)	502	5 (1.00)	293	2 (0.68)
Tanjung Rusa	119	2 (1.68)	424	2 (0.47)	399	1 (0.25)
All villages	3,024	76 (2.51; 1.99-3.14)	3,715	32 (0.86; 0.59-1.21)	3,559	39 (1.10; 0.78-1.49)

Values are presented as number positive (percentage). 95% confidence intervals are shown for total prevalence only.

### Efficacy of IDA in Mf-positive participants and compliance with MDA

**Fig 3:**
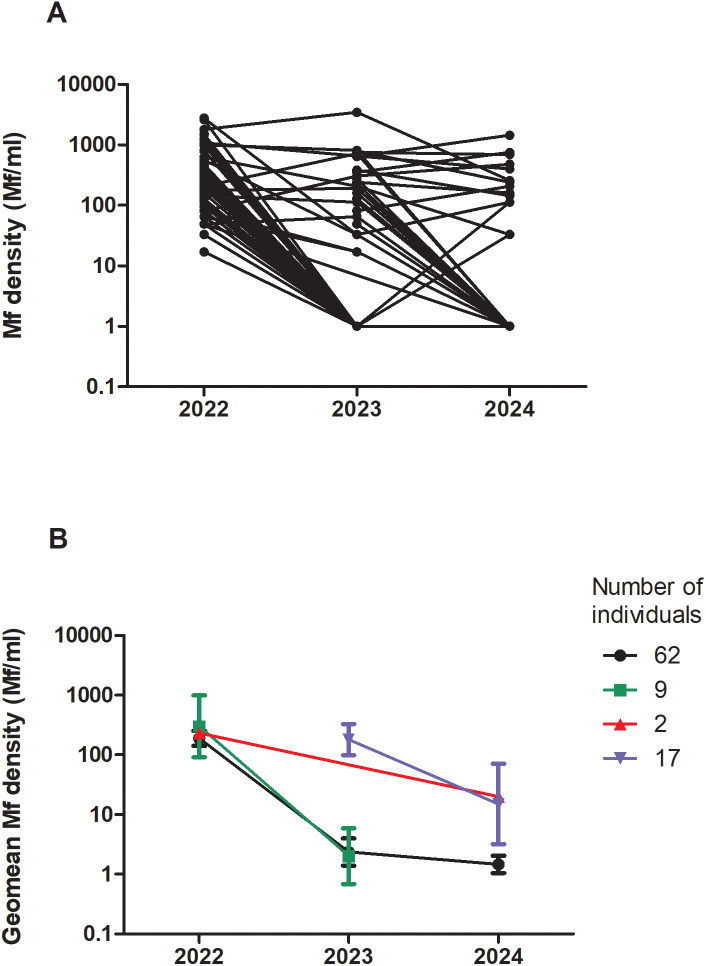
The dynamics of Mf density of participants from 2022–2024. **(A)** Mf densities of individuals before treatment (2022) and after the first and second rounds of MDA. **(B)** Geometric means of Mf density before treatment (2022) and after the first and second rounds of MDA. Black line: individuals with baseline in 2022 and follow ups in 2023 and 2024; green line: individuals with baseline in 2022 and follow up in 2023; red line: individuals with baseline in 2022 and follow up in 2024; blue line: individuals with baseline in 2023 and follow up in 2024.

The study was designed as a cross-sectional study to evaluate the impact of MDA on the Mf prevalence. In addition, we specifically followed up Mf-positive individuals from 11 villages. We detected at baseline 76 Mf-positive individuals. Following the first round of MDA (2023), there were 71 Mf-positive individuals, of which 59 (83%) became Mf-negative. After the second round of MDA (2024), we followed up a total of 90 Mf-positive persons (73 persons from 2022, and 17 Mf-positive persons who were found in 2023). Fourteen of these 90 people (15,6%) were still Mf-positive in 2024, with one person admitting to having participated in only one round of MDA, while the rest participated in two rounds of MDA. Interestingly, 2 persons who were Mf-positive at baseline and became Mf-negative during the first follow-up survey were again Mf-positive at the time of the second follow-up (Fig 3A). Both of these people reported that they had received both rounds of MDA.

Among 62 individuals that were followed twice (Fig 3B), 10 (16%) remained positive in 2023 and 5 (8%) remained positive by 2024. Of the 9 participants followed up in 2023 only, 2 (22%) remained positive. Additionally, 17 new Mf-positive cases were detected in 2023; of these, 8 (47%) remained positive in 2024. Of two returning cases (missing in 2023), one (50%) remained positive.

## Discussion

The use of IDA to eliminate LF has been increasingly adopted as recommended by WHO since 2017 [12]. Several studies have shown that the IDA triple-drug regimen is more efficacious than a two-drug regimen (DA) at rapidly clearing Mf [12,13,15,23,24]. On the other hand, the combination of ivermectin with DA did not increase the frequency and severity of adverse effects. Simulation modeling of IDA MDA for LF caused by *W. bancrofti* suggested that this approach can accelerate elimination if high compliance is achieved [25]. WHO recommended two rounds of triple-drug therapy as an alternative MDA regimen for LF elimination [12]. This regimen should be used in countries that are eligible to use DA, to catch up when previously no or few rounds of MDA with DA were provided or if MDA needs to be restarted because of unacceptably high numbers of infections discovered during TAS or post elimination surveillance. However, most studies on the efficacy and impact of IDA were focused on bancroftian filariasis while data on brugian filariasis are scarce [13,15,23]. The present study is the first to evaluate the impact of IDA MDA on zoophilic *B. malayi*.

Zoophilic *B. malayi* has been identified in several districts of Indonesia [1,7,8]. We were able to show that cats, dogs, and monkeys are naturally infected with *B. malayi* in Belitung district [26]. If the same mosquito species bites these animals and humans, parasites from animals could be transmitted to humans. This might explain persistence or recrudescence of *B. malayi* infections in humans following elimination status received after five rounds of MDA with DA. However, it is possible that LF was not actually eliminated, since most people with microfilaremia in the current study were over 50 years of age, therefore the TAS seroprevalence surveys in children conducted after DA MDA may have failed to detect persistent infections in the area. That is to say, the strategy of using children as sentinels to detect persistent infections following MDA was flawed in this setting.

This study clearly showed that two annual rounds of IDA MDA with high compliance reduced Mf prevalence from baseline. However, the overall upper CI for the average Mf prevalence was still above the target of 1% and 5 out of 11 villages had an average Mf prevalence in adults of more than 1%. The failure to reduce the Mf prevalence further with a second MDA may be explained by insufficient compliance to IDA MDA potentially by systematic non-compliance.

However, both rounds of reintroduced IDA MDA had a compliance of more than 65% and the IDA Impact Survey study approach used in this cross-sectional study was sensitive enough to detect infections in the area. Using this method, blood samples from adults who were at least eighteen years old were collected at baseline and two follow-ups. At baseline we detected 81 Mf-positive individuals among 5,161 adults. In contrast, we identified only 6 Mf-positive cases among 1,021 children. These data confirm that an IDA Impact Survey design targeting adults is more sensitive for detecting *B. malayi* infections in this endemic setting.

For IDA Impact Survey cluster selection, we used population-proportional sampling at baseline. We used geographic information and infection prevalence data from the baseline survey to select clusters for the follow-up surveys [27]. The results indicated that focusing on hotspot regions will increase the number of positive cases. As a result, the prevalence of infection after the first and second rounds of IDA treatment did not significantly change. Twenty additional Mf positive individuals were found after one round of IDA treatment. The number of new positive cases rose to 25 after the second follow-up evaluation post-IDA treatment. For people with persistent or recurrent infections, additional testing might be required to distinguish reinfection or recrudescence [28].

Although more women participated in the three surveys, men were almost 3 times more likely to be Mf-positive. The higher frequency of infections in men might be explained by the fact that men are more likely to be exposed to infection through outdoor employment in tin mining, palm oil plantations, or white pepper farming. In areas with a similar epidemiological setting like in Belitung surveys that are focusing on adult men may be more sensitive for detecting persistent infections without increasing the sample size.

Our results showed that 83% of Mf-positive individuals were free of Mf at follow-up after the first round of MDA. This confirmed the high efficacy of IDA for brugian filariasis [10,14]. However, these results showed also that not all study participants were free of Mf after MDA, and two Mf-positives were Mf-free at the first follow-up and then Mf-positive again at the second annual follow-up. This can be explained that despite their claim to have participated in MDA they actually might have not, or they might have cleared Mf but got quickly re-infected. The short prepatency period of *B. malayi* infection of only 3 months could explain rapid reinfection [29]. Behavioral and occupational risk factors may support reinfection in certain individuals. If persons become reinfected, the question is “what is the source of reinfection?”. One explanation could be that mosquitoes have taken up *B. malayi* Mf from an animal reservoir such as domestic animals (cats and dogs) and wildlife (*Macaca fascicularis*). This is supported by survey findings showing 4.1% in 291 cats, 2.4% in 41 dogs, and 13.5% in 163 monkeys harbored *B. malayi* parasites [26]. Although the highest Mf prevalence was found in wild monkeys, extensive land clearing for palm oil plantations has forced wildlife to live closer to human settlements, thereby increasing the risk of transmission [30].

Another explanation could be infected vectors from the adjacent district (Belitung Timur). Like Belitung, Belitung Timur is still endemic for LF caused by *B. malayi,* despite repeated MDA [31]. Unfortunately, MDA with IDA was poorly coordinated between both districts, and MDA coverage was low in Belitung Timur. It is possible that a combination of insensitivity of TAS based on antibodies in children, the presence of an animal reservoir in the area, and the reintroduction of infection from adjacent areas due to people’s migration are responsible for the failure to eliminate zoophilic *B. malayi*.

Our results raise the question of whether elimination of zoophilic *B. malayi* is feasible and if so, what additional intervention is needed. It is possible that *B. malayi* transmission requires both infections in both humans and animals. Thus, effective MDA with IDA in the human population might be sufficient to interrupt transmission in both humans and animals. MDA with moxidectin that has a mean half-life of 23 days and is effective against *W. bancrofti* and other filarial infections may have a prophylactic and longer-lasting effect against *B. malayi* [32,33]. Another strategy might be to provide more intensive MDA to the high-risk population (i.e., adult men). Various vector control measures might be useful such as elimination of suitable breeding sites of vector mosquitoes, indoor residual spraying, or use of long-lasting insecticide treated bed nets or curtains. Further, studies are needed that target the sustainable elimination of LF caused by zoophilic *B. malayi*.

The study also has limitations. The used IDA Impact Survey design recommended by WHO has not been previously evaluated for *Brugia* areas, and it is virtually unknown whether it is the most sensitive and cost-effective way to assess the impact of IDA MDA. Compliance with MDA was assessed during the follow-up survey 6–8 months after MDA and recall bias may have overestimated the compliance. MDA was done by community health workers, and the rate of directly observed treatment may have been lower than claimed. However, the biggest limitation was that IDA MDA was not performed at the same time in Belitung and the adjacent Belitung Timur district, and infection could have been introduced from Belitung Timur, which is also endemic for *B. malayi* [31]. Unfortunately, these limitations are common real-world field study problems, and we think they do not change the main messages of the study.

In conclusion, this study shows that the IDA Impact Survey study design together with geospatial modeling to focus on high infection risk areas was suitable to detect infections following MDA. It also shows that two rounds of IDA MDA in areas with zoophilic *B. malayi* were not sufficient to eliminate LF and that additional strategies are needed. At a minimum, areas with zoophilic *B. malayi* should be designated by the WHO as special intervention zones that need additional MDA and intensified post-MDA and post-elimination surveillance. As national LF elimination programs usually follow WHO-recommended strategies, more resources may be made available for LF elimination in special intervention zones.

## Supporting information

S1 Fig Timeline of Mass Drug Administration (MDA), Transmission Assessment Surveys (TAS), and Blood Surveys Before and After IDA MDA in Belitung District. (PDF)

S1 Table Selected villages and number of clusters for each village. (PDF)

S1 Text Geostatistical model for lymphatic filariasis. (PDF)

S2 Table Prevalence by gender and age group in the adult population at baseline and after the 1st and 2nd rounds of MDA in adults. (PDF)
