# Supplementary material for: Can mass drug administration alone eliminate lymphatic filariasis in areas of Indonesia with zoophilic Brugia malayi?
Source: PLoS Negl Trop Dis. 2026 Jul 14;20(7):e0014501. doi: 10.1371/journal.pntd.0014501 (PMC13367699; doi:10.1371/journal.pntd.0014501)
Supplement: S1 Fig — (PDF) [file pntd.0014501.s001.pdf]

Timeline of Mass Drug Administration (MDA), Transmission Assessment Surveys (TAS), and Blood Surveys Before and After MDA IDA in Belitung District

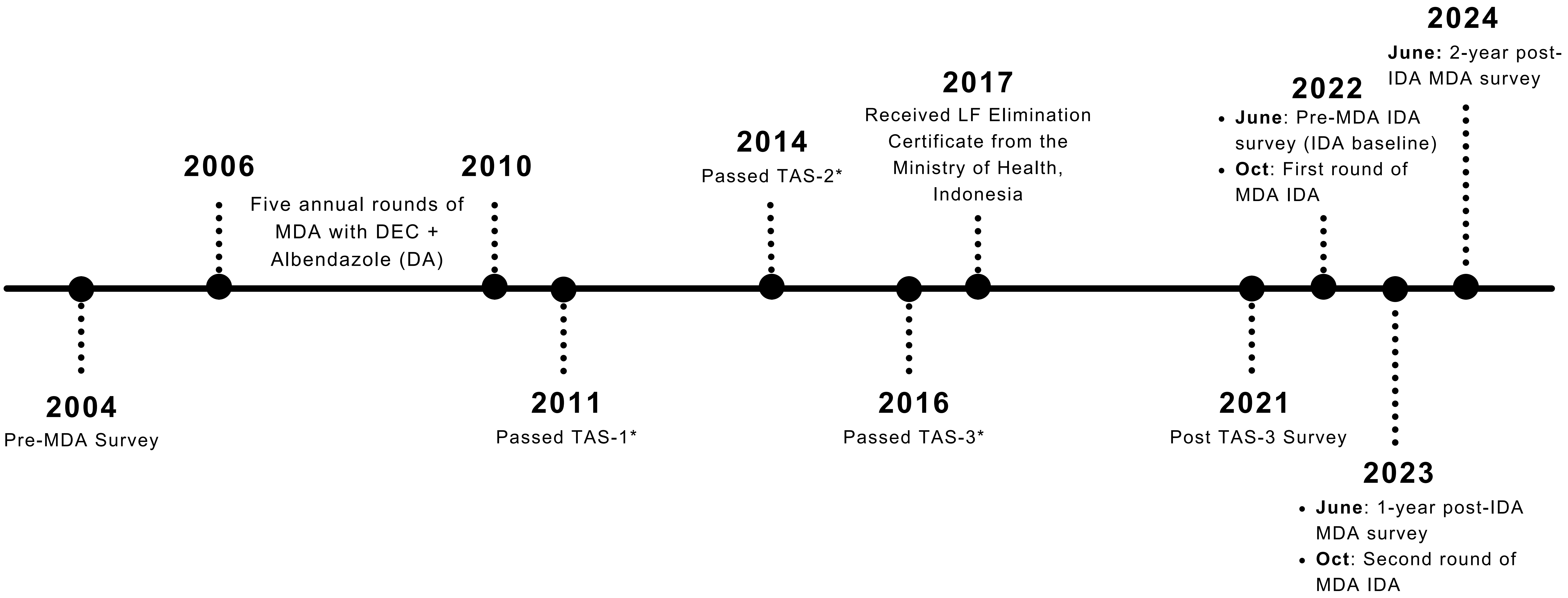

**Transmission assessment surveys (TAS) were conducted among school-aged children using anti-filarial IgG4 antibody testing.*
