# Supplementary material for: Can mass drug administration alone eliminate lymphatic filariasis in areas of Indonesia with zoophilic Brugia malayi?
Source: PLoS Negl Trop Dis. 2026 Jul 14;20(7):e0014501. doi: 10.1371/journal.pntd.0014501 (PMC13367699; doi:10.1371/journal.pntd.0014501)
Supplement: S1 Table — (PDF) [file pntd.0014501.s002.pdf]

S2 Table. Selected villages and number of clusters for each village.

Primary Health Center	Village	Coordinates	No of Clusters		
			Baseline	Post-MDA Round 1	Post-MDA Round 2
Badau	Air Batu Buding	(-2.73944666, 107.9240548)	1	1	1
	Badau	(-2.82791, 107.7877117)	2	3	3
	Cerucuk	(-2.80161689, 107.6924306)	1		
	Kacang Botor	(-2.760023833, 107.8198764)	1		
	Pegantungan	(-2.9221983, 107.5565339)		1	3
	Sungai Samak	(-2.837806667, 107.64394)	1		
Membalong	Gunung Riting	(-3.18632705, 107.7510195)	1		
	Membalong	(-3.131655183, 107.6866914)	2		
	Mentigi	(-3.188215, 107.7193183)	1		
	Padang Kandis	(-3.196443333, 107.5948617)	1		
	Perpat	(-3.06120645, 107.6414655)	1		
	Tanjung Rusa	(-3.15393325, 107.831104)	1	2	3
Sijuk	Air Selumar	(-2.61472473, 107.7503548)	1		
	Air Seruk	(-2.65357, 107.709025)	2		
	Pelepak Pute	(-2.649092567, 107.8314684)	1		
	Sijuk	(-2.569566667, 107.76886)	1		
	Sungai Padang	(-2.58526274, 107.8888531)	1	3	2
Simpang Rusa	Bantan	(-2.888130294, 107.6343235)	1	2	3
	Kembiri	(-3.056081917, 107.7724247)	1	4	2
	Lassar	(-3.030538, 107.642775)	2	6	6
	Simpang Rusa	(-2.941224767, 107.6223394)	1	4	4
Tanjung Binga	Batu Itam	(-2.69821, 107.6213233)	1		
	Keciput	(-2.573447786, 107.6782007)	1		
	Terong	(-2.624565, 107.6494517)	1		
Selat Nasik	Petaling	(-2.872969817, 107.4439242)	1	1	1
	Selat Nasik	(-2.838404983, 107.4134273)	1	2	1
	Suak Gual	(-2.917298633, 107.3874761)	1	1	1
	**Total**		**30**	**30**	**30**
