# Supplementary material for: Can mass drug administration alone eliminate lymphatic filariasis in areas of Indonesia with zoophilic Brugia malayi?
Source: PLoS Negl Trop Dis. 2026 Jul 14;20(7):e0014501. doi: 10.1371/journal.pntd.0014501 (PMC13367699; doi:10.1371/journal.pntd.0014501)
Supplement: S1 Text — (PDF) [file pntd.0014501.s003.pdf]

Supplementary Material: Geostatistical Model for Lymphatic Filariasis Surveillance in Belitung District, Indonesia

1 Introduction

This supplementary material provides a detailed description of the geostatistical model used to analyse the spatial distribution of lymphatic filariasis (LF) prevalence and to guide cluster selection for follow-up surveys in Belitung District, Indonesia.

2 Model Specification

We adopted a model-based geostatistical approach [Diggle and Ribeiro, 2007, Diggle et al., 2013] to analyse the spatial distribution of LF prevalence. Let Y_i denote the number of microfilaria (Mf)-positive individuals out of n_i tested at location x_i for $i = 1, \ldots, m$, where m is the number of surveyed villages. We assume that, conditional on a latent spatial process $S(x)$, the counts follow independent binomial distributions:

$$Y_i \mid S(x_i) \sim \text{Binomial}(n_i, p(x_i)) \tag{1}$$

where $p(x_i)$ is the prevalence of LF at location x_i.

The prevalence is modelled through a logit link function:

$$\log\left(\frac{p(x_i)}{1 - p(x_i)}\right) = \beta_0 + S(x_i) \tag{2}$$

where β_0 is the intercept representing the overall log-odds of infection, and $S(x_i)$ is a stationary and isotropic Gaussian spatial process that captures the spatial correlation in LF prevalence across the study area.

The spatial process $S(\cdot)$ is assumed to be a zero-mean Gaussian process with an exponential correlation function:

$$\text{Cov}(S(x_i), S(x_j)) = \sigma^2 \exp\left(-\frac{\|x_i - x_j\|}{\phi}\right) \tag{3}$$

where σ^2 is the variance (or sill) of the spatial process representing the overall variability in the log-odds of infection across the study area, ϕ is the scale parameter (or range) that controls the rate of decay of spatial correlation with distance, and $\|x_i - x_j\|$ denotes the Euclidean distance between locations x_i and x_j. The exponential correlation function implies that the spatial correlation decreases exponentially with distance. The practical range, defined as the distance at which the correlation drops to approximately 0.05, is given by 3ϕ [Diggle and Ribeiro, 2007].

3 Parameter estimation and spatial prediction

The binomial geostatistical model was fitted using the `PrevMap` package [Giorgi and Diggle, 2017] in the R statistical computing environment [R Core Team, 2024]. Model parameters were estimated using Monte Carlo maximum likelihood (MCML) [Christensen et al., 2004], which approximates the likelihood function through Monte Carlo simulation. The estimated parameters

include $\hat{\beta}_0$ (the estimated intercept), $\hat{\sigma}^2$ (the estimated variance of the spatial process), and $\hat{\phi}$ (the estimated spatial range parameter).

For the binomial geostatistical model specified in equations (1)-(3), spatial prediction at unsampled locations cannot be performed using standard kriging equations that apply to Gaussian geostatistical models. Let $\mathbf{Y} = (Y_1, \ldots, Y_m)^T$ denote the vector of observed Mf-positive counts at the m surveyed locations, $\mathbf{n} = (n_1, \ldots, n_m)^T$ the vector of sample sizes, $\mathbf{S} = (S(x_1), \ldots, S(x_m))^T$ the vector of the spatial process at observed locations, and $\boldsymbol{\theta} = (\beta_0, \sigma^2, \phi)^T$ the vector of model parameters. The hierarchical structure of the model implies that $\mathbf{Y}$ and $S(x_0)$ for an unsampled location x_0 are conditionally independent given $\mathbf{S}$, but marginally dependent through their shared dependence on the spatial process.

Spatial prediction for the binomial logistic model requires computing the predictive distribution of $S(x_0)$ conditional on the observed data $\mathbf{Y}$. This predictive distribution is given by:

$$[S(x_0) \mid \mathbf{Y}, \boldsymbol{\theta}] = \int [S(x_0) \mid \mathbf{S}, \mathbf{Y}, \boldsymbol{\theta}][\mathbf{S} \mid \mathbf{Y}, \boldsymbol{\theta}] \, d\mathbf{S} \tag{4}$$

where $[A]$ denotes the probability distribution of A. Because $S(x_0)$ and $\mathbf{Y}$ are conditionally independent given $\mathbf{S}$, we have $[S(x_0) \mid \mathbf{S}, \mathbf{Y}, \boldsymbol{\theta}] = [S(x_0) \mid \mathbf{S}, \boldsymbol{\theta}]$, which is Gaussian with mean and variance determined by the conditional distribution of a Gaussian process [Diggle and Ribeiro, 2007]. However, the marginal distribution $[\mathbf{S} \mid \mathbf{Y}, \boldsymbol{\theta}]$ is not Gaussian due to the binomial likelihood, making the integral in equation (4) analytically intractable.

To approximate this predictive distribution, we employ Monte Carlo methods as follows. First, we draw samples $\mathbf{S}^{(k)} = (S^{(k)}(x_1), \ldots, S^{(k)}(x_m))$ for $k = 1, \ldots, N$ from the distribution $[\mathbf{S} \mid \mathbf{Y}, \hat{\boldsymbol{\theta}}]$ using Langevin-Hastings Markov chain Monte Carlo (MCMC) methods implemented in `PrevMap`. For each sample $\mathbf{S}^{(k)}$, we then generate $S^{(k)}(x_0)$ from the conditional Gaussian distribution $[S(x_0) \mid \mathbf{S}^{(k)}, \hat{\boldsymbol{\theta}}]$, which has mean $\mathbf{c}^T(x_0)\mathbf{C}^{-1}\mathbf{S}^{(k)}$ and variance $\sigma^2 - \mathbf{c}^T(x_0)\mathbf{C}^{-1}\mathbf{c}(x_0)$, where $\mathbf{c}(x_0)$ is the vector of covariances between $S(x_0)$ and $(S(x_1), \ldots, S(x_m))$ and $\mathbf{C}$ is the $m \times m$ covariance matrix of $\mathbf{S}$. The collection $\{S^{(1)}(x_0), \ldots, S^{(N)}(x_0)\}$ provides a Monte Carlo sample from the predictive distribution $[S(x_0) \mid \mathbf{Y}, \hat{\boldsymbol{\theta}}]$.

The predictive mean of the prevalence at location x_0 is then approximated as:

$$\hat{p}(x_0) = \frac{1}{N} \sum_{k=1}^{N} \frac{\exp(\hat{\beta}_0 + S^{(k)}(x_0))}{1 + \exp(\hat{\beta}_0 + S^{(k)}(x_0))} \tag{5}$$

and similarly for other quantities of interest. For surveillance purposes, a key objective was to identify villages with LF prevalence exceeding the critical threshold $p^* = 0.01$, as this threshold has implications for WHO recommendations on continued MDA [WHO, 2017]. The exceedance probability at location x_0 is:

$$\pi(x_0) = P(p(x_0) > p^* \mid \mathbf{Y}, \hat{\boldsymbol{\theta}}) \tag{6}$$

which we approximate using the Monte Carlo samples as:

$$\hat{\pi}(x_0) = \frac{1}{N} \sum_{k=1}^{N} \mathbb{I}\left(\frac{\exp(\hat{\beta}_0 + S^{(k)}(x_0))}{1 + \exp(\hat{\beta}_0 + S^{(k)}(x_0))} > p^* \right) \tag{7}$$

where $\mathbb{I}(\cdot)$ is the indicator function.

4 Adaptive cluster selection

For the follow-up surveys conducted after the first and second rounds of IDA MDA, we implemented a risk-based sampling strategy guided by the geostatistical model predictions. This approach differs from the baseline survey, which used probability proportional to estimated size (PPES) sampling as recommended by the WHO IDA Impact Survey design [WHO, 2017].

After completing each survey (baseline and first follow-up), we applied the following systematic procedure:

1. We fitted the binomial geostatistical model specified in equations (1)–(3) to the observed prevalence data $\mathbf{Y}$ from surveyed villages, obtaining parameter estimates $\hat{\boldsymbol{\theta}} = (\hat{\beta}_0, \hat{\sigma}^2, \hat{\phi})$ using MCML as described in Section 3.

2. For each unsampled village at location x_0, we used the Monte Carlo approach from equation (4) to obtain samples from the predictive distribution $[S(x_0) \mid \mathbf{Y}, \hat{\boldsymbol{\theta}}]$ by first sampling from $[\mathbf{S} \mid \mathbf{Y}, \hat{\boldsymbol{\theta}}]$ using Langevin-Hastings MCMC, then generating conditional predictions $S^{(k)}(x_0)$ from the Gaussian conditional distribution $[S(x_0) \mid \mathbf{S}^{(k)}, \hat{\boldsymbol{\theta}}]$.

3. Using these Monte Carlo samples $\{S^{(1)}(x_0), \ldots, S^{(N)}(x_0)\}$, we computed the exceedance probability $\hat{\pi}(x_0)$ for each unsampled village according to equation (7), which quantifies the probability that prevalence at location x_0 exceeds the critical 1% threshold.

4. We ranked all unsampled villages according to their exceedance probabilities $\hat{\pi}(x_0)$, with higher probabilities indicating greater likelihood of having prevalence above the 1% threshold as defined in equation (6).

5. We selected the top 30 villages (or a subset thereof) with the highest exceedance probabilities, along with villages that had Mf-positive individuals detected in previous surveys, regardless of their exceedance probability.

Villages with high exceedance probabilities represent locations where, based on the fitted spatial process $S(\cdot)$ from equation (2) and the observed data $\mathbf{Y}$, there is substantial evidence that prevalence exceeds the critical threshold $p^* = 0.01$. The spatial correlation structure captured by the exponential correlation function in equation (3), with estimated parameters $\hat{\sigma}^2$ and $\hat{\phi}$, allows us to borrow strength from neighboring villages to make informed predictions about infection risk in unsampled locations.

References

Christensen OF, Roberts GO, Skøld M. Robust Markov chain Monte Carlo methods for spatial generalized linear mixed models. *Journal of Computational and Graphical Statistics.* 2004;13(1):1–23.

Diggle PJ, Ribeiro PJ. *Model-based Geostatistics.* New York: Springer; 2007.

Diggle PJ, Giorgi E Model-based geostatistics for prevalence mapping in low-resource settings. *Journal of the American Statistical Association.* 2013;108(504):1423–1433.

Giorgi E, Diggle PJ. PrevMap: an R package for prevalence mapping. *Journal of Statistical Software.* 2017;78(8):1–29.

Hay SI, Noor AM, Nelson A, Tatem AJ. The accuracy of human population maps for public health application. *Tropical Medicine & International Health.* 2006;11(7):1073–1086.

Hijmans RJ. raster: Geographic Data Analysis and Modeling. R package version 3.6-23; 2023.

Pebesma EJ, Bivand RS. Classes and methods for spatial data in R. *R News.* 2005;5(2):9–13.

R Core Team. R: A Language and Environment for Statistical Computing. Vienna, Austria: R Foundation for Statistical Computing; 2024.

Stein ML. *Interpolation of Spatial Data: Some Theory for Kriging.* New York: Springer; 1999.

Sturrock HJW, Gething PW, Clements ACA, Brooker S. Optimal survey designs for targeting chemotherapy against soil-transmitted helminths: effect of spatial heterogeneity and cost-efficiency of sampling. *The American Journal of Tropical Medicine and Hygiene*. 2014;82(6):1079–1087.

Waller LA, Gotway CA. *Applied Spatial Statistics for Public Health Data*. Hoboken, NJ: Wiley; 2004.

World Health Organization. Guideline: alternative mass drug administration regimens to eliminate lymphatic filariasis. Geneva: World Health Organization; 2017.

Wickham H. *ggplot2: Elegant Graphics for Data Analysis*. New York: Springer; 2016.
