# Supplementary material for: Can mass drug administration alone eliminate lymphatic filariasis in areas of Indonesia with zoophilic Brugia malayi?
Source: PLoS Negl Trop Dis. 2026 Jul 14;20(7):e0014501. doi: 10.1371/journal.pntd.0014501 (PMC13367699; doi:10.1371/journal.pntd.0014501)
Supplement: S2 Table — (PDF) [file pntd.0014501.s004.pdf]

S4 Table. Prevalence by gender and age group in the adult population at baseline and after the 1st and 2nd rounds of MDA in adults.

Age Group	Baseline (n = 26 villages)			Post-MDA Round 1 (n = 12 villages)			Post-MDA Round 2 (n = 12 villages)		
	Male (%)	Female (%)	Total (%)	Male (%)	Female (%)	Total (%)	Male (%)	Female (%)	Total (%)
18-20	1/78 (1.28)	0/87 (0.00)	1/165 (0.61)	0/72 (0.00)	1/114 (0.88)	1/186 (0.54)	0/91 (0.00)	0/111 (0.00)	0/202 (0.00)
21-30	2/365 (0.55)	2/535 (0.37)	4/900 (0.44)	1/307 (0.3)	1/402 (0.25)	2/709 (0.28)	2/332 (0.60)	1/430 (0.23)	3/762 (0.39)
31-40	8/470 (1.70)	2/692 (0.29)	10/1162 (0.86)	4/367 (1.09)	1/428 (0.23)	5/795 (0.63)	5/358 (1.40)	2/432 (0.46)	7/790 (0.89)
41-50	16/616 (2.60)	3/744 (0.40)	19/1360 (1.40)	7/455 (1.54)	3/585 (0.51)	10/1040 (0.96)	6/423 (1.42)	3/572 (0.52)	9/995 (0.90)
>50	32/773 (4.14)	15/801 (1.87)	47/1574 (2.99)	14/542 (2.58)	2/597 (0.34)	16/1139 (1.40)	19/575 (3.30)	4/599 (0.67)	23/1174 (1.96)
**Total Adult**	**59/2302 (2.56)**	**22/2859 (0.77)**	**81/5161 (1.57)**	**26/1743 (1.49)**	**8/2126 (0.38)**	**34/3869 (0.88)**	**32/1779 (1.80)**	**10/2144 (0.47)**	**42/3923 (1.07)**
